# *ssarp*: An R Package for Efficiently Inferring Species‐ and Speciation‐Area Relationships

**DOI:** 10.1002/ece3.73981

**Published:** 2026-07-08

**Authors:** Kristen M. Martinet, Cristian Román‐Palacios, Luke J. Harmon

**Affiliations:** ^1^ College of Information Science University of Arizona Tucson Arizona USA; ^2^ Bioinformatics and Computational Biology Program University of Idaho Moscow Idaho USA; ^3^ Department of Biological Sciences University of Idaho Moscow Idaho USA

**Keywords:** biodiversity, island biogeography, speciation‐area relationship, species‐area relationship

## Abstract

A universal method of examining patterns of biodiversity on islands is the species‐area relationship (SAR). SARs quantify the relationship between species richness (the number of species) and the area of the land mass on which they occur. An extension of the SAR, the speciation‐area relationship (SpAR), quantifies the relationship between speciation rate and area. Comparing these relationships across island systems globally is a difficult task because gathering and processing a large amount of species occurrence and island data often requires researchers to conduct lengthy literature searches and combine datasets from several different sources. We present *ssarp* (Species‐/Speciation‐Area Relationship Projector), an R package that provides a systematic workflow for curating data, estimating speciation rates, and inferring SARs and SpARs. The *ssarp* workflow allows users to generate SARs and SpARs using either occurrence records or presence‐absence matrices. Functions in *ssarp* use mapping tools to associate GPS points with land masses, remove points not on land, associate land masses with their areas using a built‐in dataset of island names and areas, and infer SARs using unsegmented and segmented regression. The accuracy of these records can also be assessed by the user through the creation of a presence‐absence matrix from *ssarp*'s workflow. The *ssarp* R package also provides functions for estimating speciation rates and generating a SpAR. The *ssarp* R package allows researchers to increase the scope of their biodiversity research by efficiently inferring SARs and SpARs with occurrence records and presence‐absence matrices.

## Graphical abstract

The *ssarp* (Species‐/Speciation‐Area Relationship Projector) R package provides users with a streamlined workflow for inferring species‐ and speciation‐area relationships for island‐dwelling species. The *ssarp* R package allows researchers to increase the scope of their biodiversity research by providing a systematic workflow for curating data, estimating speciation rates, and inferring species‐ and speciation‐area relationships.

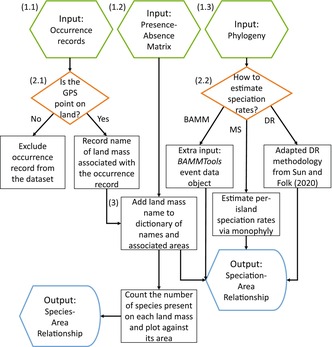

## 1 Introduction

MacArthur and Wilson's (1967) equilibrium theory of island biogeography introduced a foundational framework for understanding patterns of biodiversity on islands. This original framework proposed that island species richness (the number of species on an island) would be stable at an equilibrium point between immigration and extinction rates, with dynamics that depend on island size and isolation. The classic model of island biogeography has been expanded upon multiple times, with important additions relating to phylogenetic diversification (Heaney 2000), island ontogeny (Whittaker et al. 2008), and species abundances (Rosindell and Harmon 2013). There is now a rich body of work focusing on using species‐area relationships (SARs) and speciation‐area relationships (SpARs) to jointly analyze macroecological and macroevolutionary patterns in insular systems.

The observation that species richness increases with increasing area is a fundamental law of ecology (Arrhenius 1921; Gleason 1922; Rosenzweig 1995). A decrease in biodiversity can disrupt this relationship, which can be mediated by either natural or anthropogenic effects. For example, island ontogeny, the “life cycle” of an island, ends with a reduction of the island's area during submergence, which also reduces the size of habitat and impacts species richness through extinction (Whittaker et al. 2008). Similarly, anthropogenic effects such as habitat loss and fragmentation and increasing numbers of non‐native species negatively affect SARs (Chisholm et al. 2018; Baiser and Li 2018; Guo et al. 2021). While the foundational work for island biogeography focused on generating SARs for oceanic archipelagos, these relationships have also been applied to “island‐like” systems that share similar qualities with oceanic islands. For example, SARs have been used in lakes to assess ecosystem health (Wu et al. 2022) and in forest habitat fragments to describe ecoregion biodiversity (Steffens et al. 2025). Trends in species richness that are visualized in SARs can be further explained through the use of SpARs. In addition to uncovering relationships between speciation and area, SpARs are sometimes able to pinpoint a threshold land area for in situ speciation (Losos and Schluter 2000) or, in contrast, find that in situ speciation occurs regardless of area (Wagner et al. 2014; Schluter and Pennell 2017).

Exploring how trends in biodiversity differ between different islands and archipelagos can help explain how biodiversity is generated globally. However, global comparisons of island systems typically require a lengthy literature search and a combination of datasets from several SAR‐related papers (e.g., Baiser and Li 2018; Guo et al. 2021; da Silva et al. 2024). Further, data accessibility and reproducibility can be a barrier when conducting a literature search to compare island systems globally. For example, Baiser and Li (2018) used the *digitize* R package (Poisot 2011) to extract data from published SAR plots for use in their comparative analysis because it was not otherwise accessible. Increasing availability of occurrence data from public databases such as GBIF (Global Biodiversity Information Facility) that can be curated to inform species richness may allow researchers to more comprehensively answer questions about global biodiversity patterns without the struggles inherent in compiling data from a literature search (e.g., inaccessible data).

To date, there is currently no tool that offers a pipeline for starting with occurrence data and ultimately inferring SARs and SpARs. Researchers often access occurrence databases via their APIs using R packages such as *rgbif* (Chamberlain et al. 2024) or *spocc* (Owens et al. 2024). There are several options for filtering data using these packages, such as including only occurrence data with GPS points or restricting the geographical area of the returned data, but oftentimes this data still requires manual cleaning. The *CoordinateCleaner* R package (Zizka et al. 2019) provides a suite of useful tools for ensuring that occurrence data from web databases has meaningful GPS points for use in analyses (e.g., testing if GPS points plot in the ocean instead of on land, if locality information matches the true location of GPS points, if GPS points correspond with museum locations instead of true observations). However, the tools available with *CoordinateCleaner* mistakenly flag valid occurrence records on small islands as not on land if a buffer for the coastline is not implemented. Implementing this buffer is often tedious for islands with minimal geographic data available on global datasets. Our R package *ssarp* (Species‐/Speciation‐Area Relationship Projector) addresses the difficulties described above and makes quantifying patterns of biodiversity on a global scale more accessible by providing an intuitive pipeline that (1) combines different sources of geographic information (e.g., the *maps* R package; Becker et al. 2023) to determine whether occurrence points are truly on land and (2) allows users to input occurrence record datasets or presence‐absence matrices to infer SARs and SpARs.

## 2 Methods

The *ssarp* package first enables users to estimate species richness for taxa occurring on island systems. With these data, *ssarp* then provides functionality that allows users to fit both a linear regression model on a log–log scale (reviewed in Scheiner 2003) and a segmented regression model (reviewed in Matthews and Rigal 2021) for SARs and SpARs. Segmented regression models are constructed with utilities from the *segmented* R package (Fasola et al. 2018). While *ssarp* includes only basic functions for fitting SARs and SpARs, users can easily explore alternative approaches for analyzing outputs from *ssarp* using other tools in R. For example, the “get_richness” function in *ssarp* generates a species richness data frame that can be directly used with the *sars* R package to run a multi‐model inference approach (Matthews et al. 2019). The *ssarp* package also includes three methods for estimating speciation rates for use in generating SpARs (described below).

**FIGURE 1:**
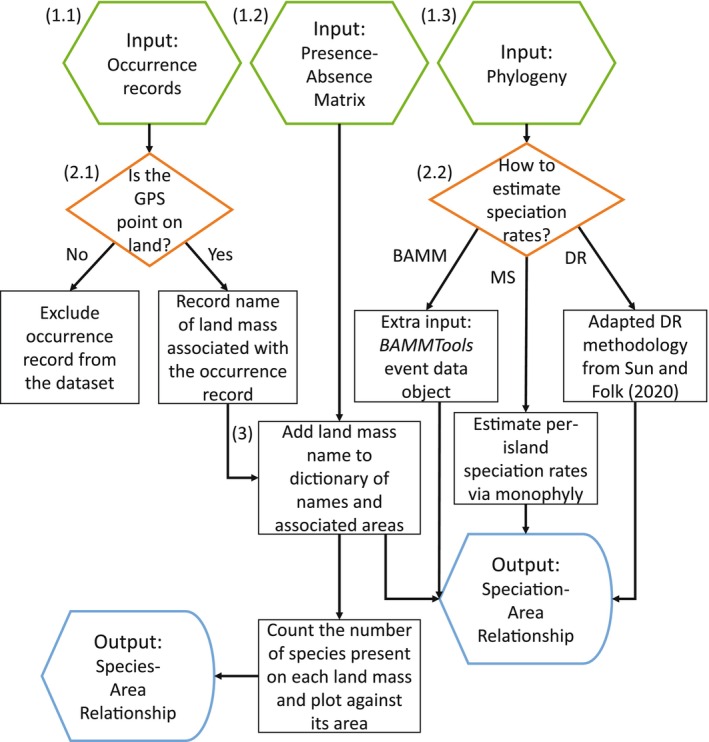
Flowchart representing the basic steps for using *ssarp* to infer a species‐area relationship (SAR) and a speciation‐area relationship (SpAR). Steps for generating a SAR are numbered as follows: Input either (1.1) an occurrence record data frame or (1.2) a presence‐absence matrix; (2.1) if occurrence records are input, determine whether the GPS points associated with the occurrence records truly correspond with land masses; (3) find the areas of those landmasses from a dataset included in the package; and infer a species‐area relationship using the resulting curated data. Steps for generating a SpAR are numbered as follows: Input either (1.1) an occurrence record data frame or (1.2) a presence‐absence matrix, along with a phylogeny (1.3); (2.1) if occurrence records are input, determine whether the GPS points associated with the occurrence records truly correspond with land masses; (3) find the areas of those landmasses from a dataset included in the package; (2.2) choose a method for estimating speciation rate; and infer a speciation‐area relationship using the resulting data.

SARs require two components: species richness data for taxa of interest and the area for each land mass on which those taxa occur. SpARs need the same two components along with a phylogeny to use in estimating speciation rates. The *ssarp* package allows users to infer species‐area relationships (SARs) for a taxon of interest (e.g., genus, family) through functions that serve as sequential steps in the SAR generation workflow (Table 1, Figure 1). Users may input either occurrence record datasets (e.g., those curated from GBIF) or presence‐absence matrices (i.e., a matrix with species names as the columns and island names as the rows, where a 1 indicates presence and a 0 indicates absence) to serve as richness data as part of *ssarp*'s workflow. To help generate SARs and SpARs for island‐dwelling species, *ssarp* also includes a built‐in dataset of island names and their associated areas that was created using global island data from Sayre et al. (2018) with ArcGIS Pro (ESRI 2024). Once occurrence data has been curated and the land mass area is recorded, the next step is to use regression models to quantify how species richness or speciation rate relates to island area.

**TABLE 1:** Functions included in *ssarp* for inferring a species‐area relationship (SAR), including aliases (where multiple names in the R package refer to the same function) in parentheses underneath each name when applicable.

Function	Description
find_land	Use mapping tools including *maps* (Becker et al. 2023) and *mapdata* (Becker et al. 2022) to find the names of land masses where occurrence records were found
find_areas	When given an occurrence record data.frame, reference a dataset of island names and areas to find areas of land masses relevant to the taxa of interest
find_pam_areas	When given a presence‐absence matrix (PAM), reference a dataset of island names and areas to find areas of land masses relevant to the taxa of interest
remove_continents	Reference a list of continental areas to remove them from the data.frame output by the find_areas function
create_sar (create_SAR)	Use unsegmented or segmented regression up to a specified number of breakpoints to generate species‐area relationship (SAR) plots. The best model as determined by AIC is returned

*Note:* We provide a brief description on their use cases as part of the SAR workflow. Figure 1 outlines the general context for using functions in this table.

To infer a SpAR for a taxon of interest, the user will follow the same workflow as with SARs, but with a user‐specified method for estimating speciation rates using a phylogenetic tree for the taxa of interest (Figure 1). Users can select a method for estimating speciation rates from three different options: BAMM (Bayesian Analysis of Macroevolutionary Mixtures; Rabosky 2014), the lambda calculation for crown groups from Magallόn and Sanderson (2001) applied to all monophyletic groups on each island, and DR (Diversification Rate; Jetz et al. 2012). In order to use the BAMM method for estimating speciation rates, the user must supply a bammdata object generated by using the *BAMMtools* R package (Rabosky et al. 2014) to read the event data file from a BAMM analysis. In order to use the Magallόn and Sanderson (2001) and the DR (Jetz et al. 2012) methods, the user must input a time‐calibrated phylogenetic tree associated with the taxa of interest. Functions specifically relevant to generating SpARs are described in Table 2.

**TABLE 2:** Functions included in *ssarp* for inferring a speciation‐area relationship (SpAR), including aliases (where multiple names in the R package refer to the same function) in parentheses underneath each name when applicable.

Function	Description
estimate_bamm (estimate_BAMM)	Use a bammdata object created using *BAMMtools* from a BAMM analysis to estimate tip speciation rates. These speciation rates are returned with the corresponding occurrence records from previous *ssarp* workflow steps. The speciation rates on each island are averaged to estimate a per‐island speciation rate.
estimate_dr (estimate_DR)	Calculate the DR statistic (Jetz et al. 2012) using methodology from Sun and Folk (2020) to estimate tip speciation rates. These speciation rates are returned with the corresponding occurrence records from previous *ssarp* workflow steps. The speciation rates on each island are averaged to estimate a per‐island speciation rate.
estimate_ms (estimate_MS)	Generate subtrees from the user‐provided phylogenetic tree and use equation 4 from Magallόn and Sanderson (2001) to estimate speciation rates for each subtree. If an island includes multiple subtrees, the island speciation rate is the average of the speciation rates of the subtrees. These speciation rates are returned with the corresponding island areas.
create_spar (create_SpAR)	Use linear (zero breakpoints) or segmented (Fasola et al. 2018) regression up to a specified number of breakpoints to infer speciation‐area relationship (SpAR) plots. The best model as determined by AIC is returned.

*Note:* We provide a brief description on their use cases as part of the SpAR workflow. Figure 1 outlines the general context for using functions in this table.

### 2.1 Finding Island Areas

Part of the workflow for *ssarp* when occurrence records are used as richness data is to determine whether a given GPS point corresponds with a land mass. The “find_land” function uses the “map. where” function in the *maps* R package (Becker et al. 2023), which returns the name of the land mass associated with a GPS point input. Multiple databases are tested sequentially in this process to attempt to fill in any gaps left over from each database reference. First, the “worldHires” database from the *mapdata* R package is used (Becker et al. 2022). Next, the “world” database from *mapdata* is used to attempt to fill in any gaps left over from using the “worldHires” database. Finally, if the “fillgaps” argument in the “find_land” function is set to “TRUE,” the Photon API (komoot 2026) will be queried for each GPS point that did not receive a land mass name from the “map. where” calls. Photon provides an easy method of accessing the OpenStreetMap API (OpenStreetMap Contributors 2026) and returns detailed information about the location associated with a GPS point. The information useful for inferring SARs for island species in *ssarp*, such as country and island name, is sometimes listed in different parts of the data returned by Photon. Considering the structure of the Photon output, “find_land” saves three sections of the Photon result: country, locality, and county. These three parameters were found to most reliably include the country and island names for a wide variety of GPS points associated with islands across the globe.

One of the most important components of *ssarp* is a dataset of island names and their associated areas. This dataset was created using *ArcPy*, a Python library for conducting geographic analyses with ArcGIS Pro (ESRI 2024). The scripts used to gather all of the island data are accessible in *ssarp*'s GitHub repository. Global island data from Sayre et al. (2018) was queried from the “Default” geodatabase in ArcGIS Pro using three separate environment masks: one for islands with an area smaller than 1 km^2^, one for islands with an area larger than 1 km^2^, and one for continents. The elevation of each island was also recorded. The “ZonalStatisticsAsTable” function was used to compile the spatial data and output it as a .csv file for use in *ssarp*. Island areas were approximated by ArcGIS Pro through the use of pixel counts. Each pixel represented a 250 m × 250 m (62,500 m^2^) area, and the reported area for each land mass was calculated by multiplying the number of pixels that cover a land mass by the area of one pixel. If users would prefer to have *ssarp* assign names and areas corresponding to a custom object, users may either input a custom data frame of island names and their associated areas, or a shapefile containing named polygons associated with the geographic areas of interest. These custom options are particularly useful for analyses relating to taxa occurring in habitat fragments or other island‐like systems.

### 2.2 Speciation Rate Estimation

Three methods for calculating per‐island speciation rates are included in *ssarp*: two tip speciation rate estimations, BAMM (Rabosky 2014) and DR (Jetz et al. 2012), and the lambda calculation with an epsilon of zero from Magallόn and Sanderson (2001) to estimate per‐island rates. The most appropriate method to use for estimating speciation rates varies based on several lineage‐specific factors (e.g., clade size, degree of rate heterogeneity). We encourage readers to consider the pros and cons of each method as applied to their study system carefully (Morlon et al. 2024). While tip speciation rate estimations, as calculated in BAMM and DR, are not useful for every analysis, examining the speciation‐area relationship (SpAR) for taxa is a good use case for tip speciation rates because these relationships focus on non‐historical geographic patterns of diversity (Title and Rabosky 2019). Users should, however, carefully consider the implications of SpARs inferred using tip speciation rates. Specifically, BAMM and DR have been criticized for inaccuracies. BAMM can give inconsistent results when run with empirical data (e.g., Meyer et al. 2018), while the DR statistic has been found to underestimate rates (e.g., Veron et al. 2025). Further, these rates do not exclusively consider diversification rates that arose on the islands considered in the SpAR analysis, but rather they incorporate the entire evolutionary history of taxa.

The “estimate_bamm” function in *ssarp* requires a bammdata object as input, which must be created using the *BAMMtools* package (Rabosky et al. 2014) after the user independently completes a BAMM analysis. This object includes tip speciation rates by default in the “meanTipLambda” list element, which *ssarp* accesses to add the appropriate tip speciation rates for each species to the occurrence record data frame. DR stands for “diversification rate,” but it is ultimately a better estimation of speciation rate than net diversification (Belmaker and Jetz 2015; Quintero and Jetz 2018) and returns results similar to BAMM's tip speciation rate estimations (Title and Rabosky 2019). The “estimate_dr” function returns the values obtained from running an adapted version of the “DR_statistic” function from Sun and Folk (2020).

In addition to tip speciation rates, *ssarp* includes a function for estimating the speciation rate for a clade using methodology from Magallόn and Sanderson (2001). The “estimate_ms” function in *ssarp* uses the “subtrees” function from *ape* (Paradis and Schliep 2019) to generate all possible subtrees from the user‐provided phylogenetic tree that corresponds with the taxa of interest for the SpAR. Then, species in the provided occurrence records generated from previous steps in the *ssarp* workflow are grouped by island. For each group of species that comprise an island, the number of subtrees that represent that group of species and the root age of each subtree are recorded, along with the name and area of the island. The diversification rate for each subtree is then calculated following equation 4 in Magallόn and Sanderson (2001). If an island includes multiple subtrees, the island speciation rate is the average of the calculated diversification rates. This average is calculated when the SpAR is plotted with the “create_spar” function.

### 2.3 Limitations of Using Global Biodiversity Data

When using occurrence records to calculate species richness, users must be cautious about using data from online databases such as GBIF. Occurrence records from GBIF used for inferring SARs using *ssarp* might need to be filtered more rigorously than the filtering mechanisms already included in the *ssarp* workflow. For example, the user might want to remove occurrence records that correspond with non‐native observations of the taxon of interest because these records might skew the resulting SAR (Baiser and Li 2018; Guo et al. 2021). These records would similarly skew resulting SpARs, especially when using the “estimate_ms” function to estimate speciation rates due to the importance of clades in the equation used in that function. Additionally, if a GPS coordinate in the occurrence dataset is dramatically incorrect, a land mass that should not be included in the taxon's range might be included in the relationship and create an outlier. Some outliers are visually obvious when the plot is generated, and faulty occurrence records can be easily spotted for removal from the data frame created by the “find_areas” function.

Beyond outliers, using GBIF records alone to infer SARs and SpARs might introduce inaccuracies due to sampling heterogeneity across archipelagos. If an island is not heavily sampled for the taxon of interest, species counts may be underestimated (Nori et al. 2023). Species are more likely to be omitted from GBIF records on islands if they are threatened or live on very small islands due to lack of sampling effort (Nori et al. 2023). GBIF also sources research‐grade observations from iNaturalist, which obscures the location for threatened species to ensure that iNaturalist is not used to poach these species. The process that iNaturalist uses to obscure these records can extend the range of these threatened species (Contreras‐Díaz et al. 2023), and in the case of oceanic island occurrence records, this method could lead to moving the GPS point into the ocean. The negative effects of this sampling heterogeneity and range expansion can be reduced by supplementing GBIF data with records from literature. We encourage users to create presence‐absence matrices using the “get_presence_absence” function in *ssarp* to carefully inspect which species are listed for each island, and edit the occurrence record data frame as necessary. Generating presence‐absence matrices from occurrence records also allows the user to conduct additional analyses, including beta diversity and other types of SARs (e.g., nested SARs). Documentation in each *ssarp* function lists requirements for custom data frames that should be referenced when supplementing GBIF data.

## 3 Applied Example

As an example of the *ssarp* workflow, we will use 10,000 records from GBIF for the lizard genus *Anolis* and infer both a species‐area relationship (SAR) and a speciation‐area relationship (SpAR) for island‐dwelling anole lizards in the Caribbean. We chose 10,000 records as the limit to minimize runtime while still generating an accurate SAR. We expect the plots generated in this example to be similar, although not exactly equivalent, to the comparison from Losos and Schluter (2000) because the Caribbean anole occurrence records we used led to slightly different values of species richness on certain islands compared to their paper. While we are focusing on GBIF records for this example, users can also input presence‐absence matrices to infer SARs and SpARs with *ssarp* (Appendix: Text A2).

### 3.1 Generating a Species‐Area Relationship

The *ssarp* package must be installed using the “install_github” function in *devtools* (Wickham et al. 2022).       devtools::install_github("ropensci/ssarp")        library(ssarp)


Once *ssarp* is installed and loaded, occurrence data can be retrieved from GBIF using *rgbif* (Chamberlain et al. 2024). While this example utilizes data from GBIF, users are able to supply their own occurrence data or a presence‐absence matrix while executing *ssarp*'s functions to infer SARs and SpARs. In order to access data from GBIF using the *rgbif* package, the key associated with the taxon of interest in the GBIF database must be determined. This key will be found using the “name_suggest” function from *rgbif*:       suggestions <- rgbif::name_suggest(query = “Anolis”, rank = “genus”)       # The key is the first element in the first row       key <- as.numeric(suggestions$data[1,1])


We will use the “occ_search” function from *rgbif* to obtain 10,000 records from GBIF for the *Anolis* genus. If the user would like to publish work that involves gathering data from GBIF to ultimately infer a SAR or SpAR with *ssarp*, we urge the user to use the “occ_download” function instead (See Appendix: Text A1 for more information). For this example, we will use the key obtained above, along with setting the “limit” parameter to 10,000 to restrict the search. The “hasCoordinate” parameter will be set to “TRUE” to ensure that all records have GPS coordinates associated with them.

Given that we are only interested in occurrence records for island‐dwelling anole lizards located in the Caribbean, we will geographically restrict the returned data to islands in this area by setting the “geometry” parameter to a polygon in Well Known Text (WKT) format that encompasses the Caribbean islands. A WKT polygon is a list of GPS points surrounding the area of interest listed in counterclockwise order. The GPS points comprising this polygon are in “longitude latitude” format with commas separating pairs.       dat <- rgbif::occ_search(taxonKey = key, limit = 10000, hasCoordinate = TRUE, geometry = 'POLYGON((-84.8 23.9, -84.7 16.4, -65.2 13.9, -63.1 11.0, -56.9 15.5, -60.5 21.9, -79.3 27.8, -79.8 24.8, -84.8 23.9))')


This data is also directly accessible as a GBIF derived dataset for reproducibility (GBIF 2025). Once the occurrence data is returned, *ssarp* will use each occurrence record's GPS point to determine the land mass on which the species was found and find the area associated with that land mass using a database of island areas and names.       land <- find_land(occurrences = dat)       area_dat <- find_areas(occs = land)


The “remove_continents” function in *ssarp* removes any continental occurrence records, which is useful when the user is only interested in island‐dwelling species. While the data obtained by using the “occ_search” function was geographically restricted, user error in specifying the polygon in WKT format might lead to accidental continental records that will be removed by using this function.       nocont_dat <- remove_continents(occs = area_dat)


The user may also run the “get_presence_absence” function from *ssarp* with the “nocont_dat” object created above to obtain a presence‐absence matrix of the species that live on each island.       nocont_pam <- get_presence_absence(occs = nocont_dat)


By running the “get_presence_absence” function with an occurrence record data frame curated by the “find_areas” function, the user will receive a data frame in the form of a presence‐absence matrix. The column names are species names, with the exception of the first column which includes island names. Within each species column, a 1 represents the presence of that species on the island corresponding to the given row, and a 0 represents the absence of that species on the island corresponding to the given row. This matrix allows the user to determine whether the occurrence data needs to be further filtered to exclude obviously incorrect records.

Next, we will generate the SAR using the “create_sar” function from *ssarp*, which creates different regression objects with breakpoints up to the user‐specified “npsi” parameter. For example, if the user inputs “npsi = 2,” “create_sar” will generate regression objects with zero, one, and two breakpoints. The function will then return the regression object with the lowest AIC score. The SAR for *Anolis* in Losos and Schluter (2000) is represented by a segmented regression with one breakpoint, so we will set “npsi” to one in this example. Note that if unsegmented regression (zero breakpoints) is better supported than segmented regression with one breakpoint, the unsegmented regression will be returned instead. The “visualize” parameter is set to “TRUE” to allow the function to automatically plot the SAR.       create_sar(occurrences = nocont_dat, npsi = 1, visualize = TRUE)


**FIGURE 2:**
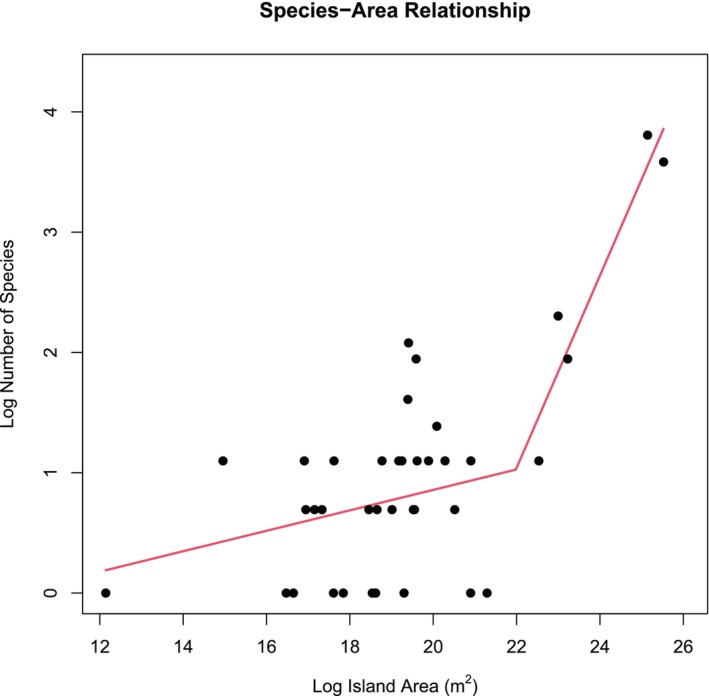
The SAR for the lizard genus *Anolis* returned by *ssarp* for the island‐based occurrences within a polygon around Caribbean islands from the first 10,000 records for the genus in GBIF. The estimated slopes for the SAR returned by *ssarp* were 0.09 and 0.71, with a breakpoint of approximately 22 log(m^2^). In comparison, the estimated slopes for the *Anolis* SAR reported by Losos and Schluter (2000) were 0.06 and 0.76, with a breakpoint of approximately 22 log(m^2^).

The “create_sar” function plots the SAR and returns a summary of the best‐fit regression model. This returned SAR is presented in Figure 2, and is very similar to the SAR reported in Losos and Schluter (2000).

### 3.2 Generating a Speciation‐Area Relationship

The “nocont_dat” object created above to generate the SAR in Figure 2 can be combined with a time‐calibrated phylogeny to infer a SpAR. This step in the *ssarp* workflow enables the user to determine whether the breakpoint in the SAR corresponds with a threshold for island size at which in situ speciation occurs (see Losos and Schluter 2000). The phylogenetic tree for *Anolis* used by Patton et al. (2021) was trimmed to only include anoles found on Caribbean islands for this example. This trimmed tree is available in *ssarp*'s GitHub repository and in the *ssarp* package's “extdata” folder. The SpAR presented in Losos and Schluter (2000) was generated using a speciation rate estimation method for each monophyletic island group that is similar to equation 4 in Magallόn and Sanderson (2001), so we will use the “estimate_ms” function from *ssarp* to estimate speciation rates in this example. The “label_type” parameter in the “estimate_ms” function corresponds to how the tip labels are written in the user‐provided phylogenetic tree. If the tip labels are simply the species epithet, as they are in the example tree here, the “label_type” parameter should be set to “epithet.” If the tip labels include the full species name, the “label_type” parameter should be set to “binomial.”       tree <- ape::read.tree(system.file("extdata", "Patton_Anolis_trimmed.tree", package = "ssarp"))       speciation_occurrences <- estimate_ms(tree = tree, label_type = “epithet”, occurrences = nocont_dat)


The newly created “speciation_occurrences” object is a data frame containing island areas with their corresponding speciation rate as estimated by the “estimate_ms” function. Next, we will use the “speciation_occurrences” object with the “create_spar” function to generate a SpAR. We will set the “npsi” parameter to one because the SpAR presented in Losos and Schluter (2000) has one breakpoint.      create_spar(occurrences = speciation_occurrences, npsi = 1, visualize = TRUE)


**FIGURE 3:**
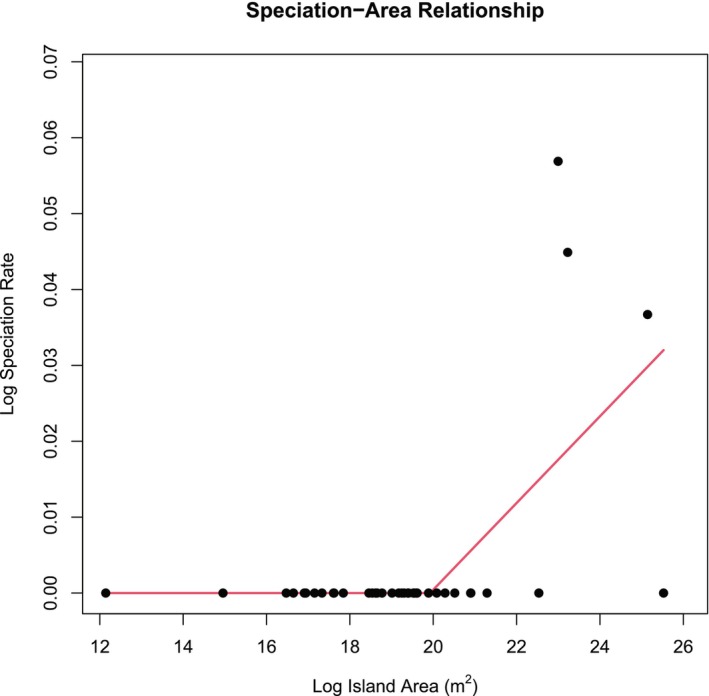
The speciation‐area relationship (SpAR) for the lizard genus *Anolis* returned by *ssarp* for the island‐based occurrences within a polygon around Caribbean islands from the first 10,000 records for the genus in GBIF. The estimated breakpoint for the SpAR returned by *ssarp* was 19.9 log(m^2^). The breakpoint for the *Anolis* SpAR reported by Losos and Schluter (2000) was approximately 22 log(m^2^). Unlike the results from Losos and Schluter (2000), the breakpoint estimated by *ssarp* for this SpAR does not match the SAR breakpoint. This likely occurred because the calculation for speciation rate in Magallόn and Sanderson (2001) that “estimate_ms” uses is based on monophyly, which can be disrupted on islands with non‐native species occurrence records.

The “create_spar” function plots the SpAR and returns a summary of the regression model. The returned SpAR for the same island‐dwelling *Anolis* data we gathered for the SAR is presented in Figure 3.

## 4 Future Directions

The current version of the *ssarp* package allows users to choose from three methods to estimate speciation rates when inferring a SpAR. There are several other popular methods for estimating speciation rates that could be added to *ssarp*, including state speciation and extinction models like GeoSSE (Geographic State Speciation and Extinction; Goldberg et al. 2011), MuSSE (Multistate Speciation and Extinction; FitzJohn 2012), HiSSE (Hidden State Speciation and Extinction; Beaulieu and O'Meara 2016), and MiSSE (Missing State Speciation and Extinction; Vasconcelos et al. 2022). Additionally, methods for inferring the number of in situ speciation events (i.e., speciation occurring within an island) for a given landmass would allow users to focus on endemic species in SpARs. Focusing on in situ speciation events would describe the evolutionary history of an island system specifically, instead of potentially incorporating the evolutionary history of colonizing ancestors through other methods of estimating speciation rates.

The *ssarp* package currently focuses on extant diversity on island systems. An additional focus on the temporal aspects of biodiversity on island systems would strengthen *ssarp*. For example, restricting speciation rate analyses to only clades that are young enough to have speciated on a particular system based on its geological age would allow users to filter out the evolutionary history of colonizing ancestors. This approach is similar to the in situ methodology described above. Incorporating more specific aspects of geological history (e.g., island ontogeny) in SARs and SpARs would provide users with additional context to describe the distribution of biodiversity on a given system. Future *ssarp* functions will also allow users to explore how changes in species richness over time impact SARs and SpARs. Finally, we encourage users to edit the island area database included in *ssarp* to ensure that it maintains accuracy. Details on how to contribute to the package are outlined on *ssarp*'s documentation website.

## 5 Conclusions

The *ssarp* package provides users with a systematic workflow for inferring species‐area relationships (SARs) and speciation‐area relationships (SpARs) using either occurrence data or presence‐absence matrices. The *ssarp* workflow increases the utility of occurrence records from online occurrence databases by providing tools for data curation (e.g., ensuring that none of the records are in the ocean) and standardization (e.g., creating a presence‐absence matrix for downstream analyses). Creating SARs and SpARs with species richness data is also streamlined with the *ssarp* package due to the inclusion of multiple functions for estimating speciation rates and generating regression objects. While the process of generating SARs and SpARs in *ssarp* is built on robust methods, outliers might still emerge. Researchers should examine resulting plots carefully to ensure that none of the occurrence data represents land masses or taxa that should not be included in the final relationship when considering the study design. The ease with which researchers infer SARs and SpARs using *ssarp* will allow for the emergence of more studies that compare these relationships using global datasets, which will hopefully lead us to a clearer picture of the world's biodiversity.

## Author Contributions

**Kristen M. Martinet:** conceptualization (lead), data curation (lead), formal analysis (lead), investigation (lead), methodology (lead), software (lead), validation (lead), visualization (lead), writing – original draft (lead). **Cristian Román‐Palacios:** methodology (supporting), project administration (supporting), resources (supporting), supervision (supporting), validation (supporting), writing – review and editing (equal). **Luke J. Harmon:** conceptualization (supporting), methodology (supporting), project administration (lead), resources (lead), supervision (lead), validation (supporting), writing – review and editing (equal).

## Funding

K.M.M. was supported by the University of Idaho Institute for Interdisciplinary Data Sciences (IIDS), Bioinformatics and Computational Biology Program (BCB), and Department of Biological Sciences.

## Conflicts of Interest

The authors declare no conflicts of interest.

## Data Availability

The R package described here is openly available on GitHub (https://github.com/ropensci/ssarp) and Zenodo (https://doi.org/10.5281/zenodo.20724047), and the data used for the Applied Example is openly available as a GBIF Derived Dataset (https://doi.org/10.15468/dd.44gngy).

## Appendix A

### Text A1: Proper GBIF Data Citation Using Derived Datasets

When using data from GBIF to create species‐area relationships (SARs) and speciation‐area relationships (SpARs), it is important to consider how to properly cite data sources. If a user downloads occurrence records for a taxon from GBIF, those records may be compiled from several different sources, each of which would need to be appropriately acknowledged. When using the “occ_search” function from *rgbif* (Chamberlain et al. 2024) to gather occurrence records, the final dataset used in the creation of a SAR or SpAR should be made available via GBIF's Derived Datasets feature. A Derived Dataset is a citable record with a DOI of the occurrence records used in your analysis and is most appropriate for citing datasets that have been heavily filtered from the original dataset download. If the “occ_download” function from *rgbif* is used to gather occurrence records, GBIF automatically creates a record of your downloaded dataset with an associated DOI for proper citation in publications that utilize this data.

In order to properly create a Derived Dataset for indexing on GBIF, the user must supply GBIF with dataset keys and occurrence record counts for each key present in the dataset. The “get_sources” function in the *ssarp* package allows the user to input an occurrence record data frame created from *ssarp*'s pipeline in order to output a data frame that includes a row for each unique dataset key and the number of occurrence records associated with each dataset. This data frame should be used, in conjunction with a DOI associated with the dataset itself uploaded on Zenodo or similar data repository, to create a Derived Dataset on the GBIF website.

### Text A2: Using Presence‐Absence Matrices to Infer SARs and SpARs

The Applied Example in the main text focused on curating GBIF data to infer a SAR and SpAR for *Anolis* lizards living on islands in the Caribbean. The *ssarp* package can also infer SARs and SpARs through the use of presence‐absence matrices. In a presence‐absence matrix (PAM), the column names are species names and the row names are island names. Within each species column, a 1 represents the presence of that species on the island corresponding to the given row, and a 0 represents the absence of that species on the island corresponding to the given row.

Using a PAM for species richness data in the *ssarp* workflow allows the user to skip the data curation steps discussed in the main text. The following code reads an example PAM that is built into the *ssarp* package. Then, it uses that PAM as an input to the “find_pam_areas” function to generate the same style of data frame that the “find_areas” function created in the Applied Example in the main text.       # Load ssarp       library(ssarp)        # Read PAM file       pam <- read.csv(system.file("extdata", "example_pam.csv",                                  package = "ssarp"))       # Create area dataframe       areas <- ssarp::find_pam_areas(pam = pam)

The “areas” data frame created above can then be used with the “create_sar” function to generate a SAR or a SpAR with the “create_spar” function (once speciation rates have been estimated).       create_sar(occurrences = areas, npsi = 1, visualize = TRUE)
